# Screening for celiac disease in juvenile idiopathic arthritis

**DOI:** 10.1186/s12969-022-00694-7

**Published:** 2022-05-12

**Authors:** Dimitri Poddighe

**Affiliations:** 1 School of Medicine, Nazarbayev University, 010000 Nur-Sultan, Kazakhstan; 2 Clinical Academic Department of Pediatrics, National Research Center for Maternal and Child Health, University Medical Center, 010000 Nur-Sultan, Kazakhstan

**Keywords:** Juvenile idiopathic arthritis, Celiac disease, HLA-DQB1, Screening, Prevalence

Dear Editor,

We read with great interest the article by Naddei et al. investigating the prevalence of Celiac Disease (CD) in a retrospective cohort of 321 patients with Juvenile Idiopathic Arthritis (JIA). Indeed, in addition to suggesting a more severe clinical course in JIA patients diagnosed with CD, they observed a 2.4% CD prevalence in their JIA cohort, which corresponds to “a 2.58-fold increased prevalence of CD compared to the general population” with respect to their study period (2001–2019) in Italy [1].

Very recently, our group reviewed all the available clinical studies investigating CD among children affected with rheumatic disorders, including JIA. Notably, we retrieved 30 CD cases in a pooled population consisting of 1174 JIA patients, which corresponds to a 2.6% prevalence of CD in JIA population [2]. This estimation is very similar to that emerging from the study by Naddei et al.

Moreover, we suggested that such a prevalence might be even underestimated, because some JIA patients positive for anti-tissue transglutaminase IgA (anti-tTG IgA) and/or anti-endomysium (EmA) antibodies, did not undergo or declined the upper gastrointestinal endoscopy (and related duodenal biopsy), which may have led to some additional CD diagnoses. We also noticed that the CD serological screening approach was quite heterogeneous across the 14 clinical studies that we analyzed and pooled, which may have further impacted on the identification of a few additional CD cases in JIA patients [2]. Indeed, as emphasized by Naddei et al. too, most patients were diagnosed with CD after the JIA onset and, thus, were “asymptomatic” (for CD), which further highlights the importance of the serological screening in patients previously diagnosed with JIA [1, 2]. These authors clearly described their follow-up strategy in JIA patients: “CD screening was systematically carried out by measuring serum anti-tTG IgA antibodies at the time of JIA diagnosis and thereafter annually. Total IgA levels were assessed at diagnosis to exclude IgA deficiency” [1]. However, as mentioned in our review, there is no (clear) recommendation for CD screening in JIA patients according to the recently updated ESPGHAN (European Society for Pediatric Gastroenterology, Hepatology and Nutrition) guidelines for CD diagnosis and management [2, 3].

Notably, such a methodological approach of complete and annual serological screening could be relatively expensive, and this may be the reason why Naddei et al. concluded their article by stating that “Future studies will test whether a first-line genetic testing followed by CD specific serological screening would be more effective than a first-line serological screening” [1]. This is not the current approach recommended by the ESPGHAN, of course; however, in our opinion too, this suggestion may deserve attention and further consideration. Indeed, we previously proposed the opportunity to consider a first-step genetic screening assessing the carriage of CD predisposing HLA-DQB1 alleles (namely, HLA-DQB1*02 and HLA-DQB1*0302) in those patients with CD first-degree relatives and/or affected with comorbidities epidemiologically associated with CD. Then, patients at risk for CD, who also result to be HLA-predisposed to CD, should undergo the (second step) periodic serological screening [4, 5].

In conclusion, in agreement with Naddei et al., we think the current evidence may support the indication to CD screening in JIA patients; however, further studies are needed to identify an appropriate and cost-effective strategy for this purpose.

## Data Availability

Not applicable.
